# International differences and inaccuracies in the public advertising about calcaneal apophysitis: an audit of websites originating in Australia, UK and USA

**DOI:** 10.1186/s13047-023-00637-9

**Published:** 2023-06-20

**Authors:** Sue Liu, Cylie M. Williams, James J. Welch

**Affiliations:** 1 Monash University, Faculty of Medicine, Nursing and Health Sciences, Wellington Road, Clayton, VIC 3168 Australia; 2 Monash University, School of Primary and Allied Health, 47-49 Moorooduc Hwy, Frankston, Vic 3199 Australia; 3 Ablefeet Ltd, 16 Terrace Road, Walton-On-Thames, Surrey, KT12 2SB UK

## Abstract

**Background:**

Calcaneal apophysitis is a common condition in childhood. Parents often seek online information for children’s’ health care concerns prior to seeking care. Therefore, we aimed to evaluate the credibility, readability, and accuracy of calcaneal apophysitis advertising on popular websites in three countries.

**Methods:**

We used content analysis of publicly accessible data. This involved identifying the top 50 websites in each country from their hit rates. We used elements of validated tools to audit and determine frequencies relevant to credibility (e.g. publisher), readability (e.g. literacy score) and accuracy (e.g. alignment with evidence). Data were analysed quantitatively and reported against each element.

**Results:**

Websites were predominantly hosted by private health services (*n* = 118, 79%). The mean (SD) SMOG (readability) score was 9.3 (4.5). The majority of websites (*n* = 140, 93%) provided at least one treatment recommendation, and less than 10% (*n* = 11) of websites advertised treatments fully aligned with evidence. Use of treatment modalities without evidence and with high risk to children were also found including surgery, extracorporeal shock wave therapy and laser.

**Conclusions:**

Calcaneal apophysitis online advertising is mostly curated by clinicians. Clinicians should consider revising online advertising to increase understandability and accuracy to reduce health care wastage, risk, and low value care.

**Supplementary Information:**

The online version contains supplementary material available at 10.1186/s13047-023-00637-9.

## Background

Parents commonly use online information to determine when, and who to see for their children’s health conditions [1]. However, despite finding information easy to understand, there are concerns with navigating the accuracy of health condition treatment advertising or information [2]. Evidence-based condition information can improve health outcomes and reduce excessive costs to both families and healthcare services. For example, online information can provide self-treatment options, or when to seek personalised advice from healthcare professionals. Online information can also provide opportunities for healthcare providers to market the services they offer.

However, online condition-specific information and treatment advertising is not always consistent with evidence-based practice [3]. Inaccurate information can drive unnecessary fear, inappropriate use of health services [4] and an increase in hospital or private practice consultations where home-based care could first be initiated in the acute phase. Calcaneal apophysitis is a common childhood condition with a 0.0037 incidence per person year in the general child population [5, 6]. Calcaneal apophysitis is frequently known by its eponymous term, Sever’s Disease, and presents with a higher incidence in children who play sport [5, 7]. Parents often seek care for their child with calcaneal apophysitis from a broad range of primary health professionals such as family medical practitioners, orthopaedic or sports medicine doctors, podiatrists, or physiotherapists. Children may also present to hospital outpatient departments in countries with limited community-based health care options [8].

At present, calcaneal apophysitis is commonly considered benign and self-resolving in most children [9]. This means good quality health advice may prevent health care wastage in both primary care and hospitals. Therefore, the primary aim of this research was to evaluate the credibility, readability, and accuracy of calcaneal apophysitis information (including diagnosis and treatment options) advertised on most frequently visited websites in Australia, the United Kingdom (UK) and United States of America (USA). The secondary aim was to descriptively explore any difference between these countries. These countries were chosen based on their models and funding of health services being predominantly privatively funded (USA), mixed public and privately funded (Australia), and predominately public funded (UK).

## Methods

### Design

The study design included content analysis of publicly available information provided about children’s heel pain consistent with calcaneal apophysitis. This design is a systematic method of coding text in order to categorise similar words and sentences [10].

### Procedure

We were initially supported in the development of search terms by five non-health professional parents. We asked these parents to describe the terminology they would use when searching for health care advice if their child had heel pain. Parents preferred online search terms were “child heel pain” or “my child has heel pain”.

Next, we used scraping software (Thruuu by app.samuelschmitt.com) in September 2021. We used this software to extract the top 300 location specific websites (top 100 in each region of interest) through their organic ranking by Google search engine results page analysis. This software program uses specific keywords entered by the user to identify websites with the search terms and extracts both the webpage URL from Google and key information from that website. This method of data acquisition and software program was chosen to eliminate influence of website choice through any saved settings, location blockers or “cookies” on authors computers. This method of data acquisition was also chosen to minimise the impact the influence of paid advertising where possible, on the ranking of the website. We used the terms “child” “heel” “pain” as three separate words in the scraping program in addition to limit country of origin to USA, UK and Australia. It was pre-determined to exclude websites only describing children’s heel pain presentations inconsistent with calcaneal apophysitis (e.g., inflammatory disease), and exclusion occured during manual data extraction by research team. Where a site was excluded based on its content, we moved to the next ranked site on the extraction list until we had 50 sites extracted for each country.

Next, the data were extracted by the scraping program in the form of an excel spreadsheet. This extracted data included the website link and organic ranking. The scraping program provided additional information such as number of images and word count. All other website information was manually extracted by the author team in a purpose-built spreadsheet to minimise the influence of any online advertising that would potentially change the webpage content in the scraping program’s extraction.

The first 50 websites provided from the Thruuu extraction of each geographical search were manually checked by all authors for location matching. Where one was outside the location, an additional was included until we had a final 150 websites (50 from each geographic location) to audit. All additional data were extracted by one author and checked by a second author (USA = JJW and CMW, UK = SL and CMW, Australia = SL and JJW). As two authors (JJW, CMW) are practicing health professionals in the UK and Australia, JJW and CMW did not audit or secondary check websites arising from the country they worked in to minimise bias.

We extracted information into three domains: credibility, readability, and accuracy of online advertising information. Our created purpose-built spreadsheet used elements from the DISCERN tool [11] and JAMA instrument [12] designed to describe or measure quality of patient information into the categories of credibility, readability and accuracy. Both tools are designed predominantly for written information and when there are published treatment guidelines to assess against. As there is no published diagnostic or treatment guidelines for calcaneal apophysitis, the questions were not applicable, therefore we applied the principles from both into the purpose-built spreadsheet. Therefore, the quality of patient information aligning with these tools included the credibility domains relating to publisher information and date of publication. Readability information first calculated using the Statistical Measure of Gobbledygook (SMOG) score, number of words and percentage of complex words. This was performed by cutting and pasting the entire information from the website into an open access SMOG online readability calculator (https://www.webfx.com/tools/read-able/). The SMOG score is calculated by the number of words containing three or more syllables within three passages of ten sentences or more [13]. The output is an estimate of the number of years of education a person requires to understand the text. This online calculator also determined the number of words and the percentage of complex words (polysyllabics, with three or more syllables). Lastly, collation of accuracy relating to the condition-related data were developed using an inductive coding framework [14]. In this section, our team built the initial framework through adding elements where new ones arose. As the framework evolved, we returned to previously extracted data and recoded as appropriate. We then used published systematic reviews and clinical trials to determine if there was evidence supporting each of the statements. The final purpose-built extraction elements are outlined in Additional file 1.

Data extracted was described in frequencies, means and standard deviations (SD). There are no consensus-based methods for the diagnosis of calcaneal apophysitis. Research commonly advocates and supports clinicians to diagnose calcaneal apophysitis based on its signs and symptoms through history taking, pain on palpation consistent with location of apophysis at the heel, and in the absence of any other localised inflammation or joint pain as a potential indicator of systemic inflammation [15, 16]. Webpages describing these signs and symptoms were considered aligned with evidence. It is also commonly accepted that medical imaging is not sensitive or specific for apophysitis diagnosis [17, 18]. Based on this we considered recommendations for additional tests and images to diagnosed calcaneal apophysitis to not be aligned with evidence.

Once we extracted the treatment elements, we aligned them with treatment groupings as described below. The treatment elements were extracted from the most recently published systematic review [19] and randomised clinical trials (cross over effect, treatment A versus placebo, treatment A versus treatment B, or a wait and see methodology) [20–24] found through a forward chaining search on Google Scholar using the most recent systematic review as the content source as at 17^th^ of September, 2021 [19]. We then grouped these treatments through the proposed mechanism for action. These groupings were developed with an experienced group of seven clinician researchers with experience in apophyseal injury management external to this present research. These clinical researchers were physiotherapists (*n* = 4) and podiatrists (*n* = 3) all having greater than 10 years extensive clinical and research experience relating to lower limb apophyseal conditions. We considered evidence supporting use, to be treatments studied in randomised control trials or quasi-randomised control trials [20, 21]. If we were unable to find any clinical trials examining the intervention, or they were studied without evidence supporting effectiveness, we grouped the treatments as having no known evidence supporting use for the condition.

Therefore, the extracted treatments groups were:

1. Those treatment groupings with evidence support through randomised control trials supporting use for this condition: load reduction strategies [20] exercise focused on building strength (eccentric) [20], rest or reassurance about the benign nature of this condition [20], orthoses/bracing/taping for force distribution [21–23], footwear or heel cushioning [21], or heel lifts [21, 24].
2. Those treatment grouping without evidence supporting use for this condition identified by authors through the systematic review and subsequent clinical trials: pharmaceutical interventions, stretching, manual therapy (e.g. massage, foam roller), other mechanical or electrotherapy treatment modalities such as extracorporeal shock wave therapy or laser, surgery, weight management

The accuracy of diagnostic methods and treatments were coded as i) All advertised recommendations supported by evidence, ii) More advertised elements supported by evidence than not, iii) Equal number of advertised elements supported and not supported by evidence iv) More advertised elements without support of evidence than those with v) All advertised elements inconsistent with evidence. This grouping approach has been broadly employed in another study investigating advertising online treatment recommendations [25].

## Results

The full list of 150 domains are provided in Additional file 2. Websites hosted by private services including health professionals, group healthcare clinics and profession specific peak bodies were the predominant source of information for all countries (*n* = 118, 79%). Table 1 presents information relating to information credibility. There were a limited number of government or public health service websites (*n* = 31, 21% of the 150 websites), with Australia having the least number of public health entities appearing in the top-rated sources (*n* = 4, 8% of 50 Australian websites). The “other” category contained a UK newspaper article written by a journalist. Half of the 150 websites (*n* = 75) provided a publication date, and of these, 38% of the 150 websites (*n* = 57) were published within the last five years.

**Table 1:** Credibility of sources of online calcaneal apophysitis

	Total N (%)	Australia n (%)	UK n (%)	USA n (%)
**Publisher**	*N* = 150	*n* = 50	*n* = 50	*n* = 50
* Government/Public Health Service/ Hospital*	31 (21%)	4 (8%)	14 (28%)	13 (26%)
* Health professional/ group private practice/peak body*	118 (79%)	46 (92%)	35 (70%)	37 (74%)
Other*	1 (< 1%)	0 (0%)	1 (2%)	0 (0%)
**Publication date**	*N* = 150	*n* = 50	*n* = 50	*n* = 50
* Publication or review date present*	75 (50%)	26 (52%)	25 (50%)	24 (48%)
* Published or reviewed in last 5 years*	57 (38%)	20 (40%)	22 (44%)	15 (30%)
**Single health profession authoring information**	*N* = 101	*n* = 23	*n* = 31	*n* = 47
* Medical health professional*^*a*^	19 (19%)	1 (4%)	4 (13%)	14 (30%)
* Podiatrist*	60 (59%)	18 (79%)	12 (39%)	30 (64%)
* Physiotherapist*	18 (18%)	4 (18%)	13 (42%)	1 (2%)
* Other Health Professional*^*b*^	4 (4%)	0 (0%)	2 (6%)	2 (4%)

^*^Journalist

^*a*^Sports physicians, Rheumatologists, Family doctors or general practitioners, Orthopaedic surgeons with or without paediatric subspecialities

^*b*^Nurses, physician assistants, osteopath and sports therapist

There were 101 of the 150 websites produced by a single profession author or group practice of the same profession. The podiatry profession made up the majority of authorship (*n* = 60, 58% of 101 single author websites), followed by medical health professionals (*n* = 19, 19% of 101 websites) and physiotherapists (*n* = 18, 18% of 101 websites). The distribution of professions providing information differed across countries.

Table 2 provides information on the readability of information provided about children’s heel pain that were consistent with calcaneal apophysitis. All three countries had similar SMOG scores with the mean (SD) SMOG score of 9.3 (4.5), aligning with a reading level of grade 9 or age 14–15 years. The UK had the greatest variability in SMOG score, ranging from grade 5.6 or age 10–11 years to grade 13 or age 18–19 years. There were 17 websites with scores less than seven, corresponding to the top end of the age when this condition would present during childhood. The number of words and percentage of complicated words were similar across the three countries.

**Table 2:** Readability and content accuracy of online health information

	Total *Mean (SD)* or n (%)	Australia *Mean (SD)* n (%)	UK *Mean (SD)* n (%)	USA *Mean (SD)* n (%)
SMOG score	9.3^*a*^ (4.5)	9.2 (1.6)	9.9 (7.6)	8.6 (1.7)
Number of words	605.7 (363.7)	602.7 (358.5)	552.9 (256.6)	661.4 (450.6)
Percentage of complicated words	12.3 (4.11)	12.3 (2.63)	12.6 (4.84)	11.9 (4.62)
Naming conventions: Prevalence				
*Sever’s or Sever’s Disease*	143 (59%)	49 (56%)	48 (63%)	45 (60%)
*Calcaneal Apophysitis*	99 (41%)	39 (44%)	28 (37%)	30 (40%)
First naming conventions used on website	*N* = *147*	*n* = *49*	*n* = *47*	*n* = *46*
* Sever’s or Sever’s Disease*	120 (84%)	42 (86%)	44 (95%)	34 (74%)
* Calcaneal Apophysitis*	22 (16%)	7 (14%)	3 (64%)	12 (26%)
Pain location consistent with calcaneal apophysitis location when author discussion apophysitis	77 (51%)	24 (48%)	34 (68%)	18 (36%)
Apophysitis attributed to compression/ground reaction forces/stress	74 (49%)	27 (54%)	19 (38%)	27 (54%)
Apophysitis attributed to traction from Achilles tendon	71 (47%)	32 (64%)	5 (10%)	33 (66%)
Apophysitis attributed to increased activity	93 (62%)	32 (64%)	12 (24%)	23 (43%)
Described pain relating to apophysitis being subsequent to activity	71 (47%)	31 (62%)	12 (24%)	27 (54%)
Described apophysitis as benign or self-limiting	67 (45%)	20 (40%)	27 (54%)	19 (38%)
Described other conditions that may mimic calcaneal apophysitis	30 (20%)	5 (10%)	6 (12%)	19 (38%)
Frequency of different age ranges	36	18	21	18

^*a*^19 websites had a SMOG of less than 7, 45 websites had a SMOG of 8 or less

Table 2 also provides a summary of information accuracy. Children’s heel pain was commonly described on websites as Sever’s disease (*n *= 143, 59% of 242 naming conventions). Sever’s disease was only mentioned first (*n* = 120, 85%) in the information, particularly in the UK (93.6%) compared to the other countries. There were eight websites that did not mention either Sever’s disease or calcaneal apophysitis, only discussing children having heel pain. Despite this, the information provided was consistent with calcaneal apophysitis and therefore remained within the analysis.

Approximately half of the websites described the correct anatomical location being the posterior heel (*n* = 77, 51% of 150 websites), while others described locations of plantar heel or above the heel. Some websites described theories commonly accepted as being contributing factors to the development of calcaneal apophysitis. These were compression forces from ground impact, traction from the Achilles tendon and increased activity (*n* = 74, 49%, *n* = 71, 47% and *n* = 93, 62% respectively). Just under half (45%) described the condition as benign or self-limiting in nature; this description was less frequent in USA websites (38%).

**Fig. 1:**
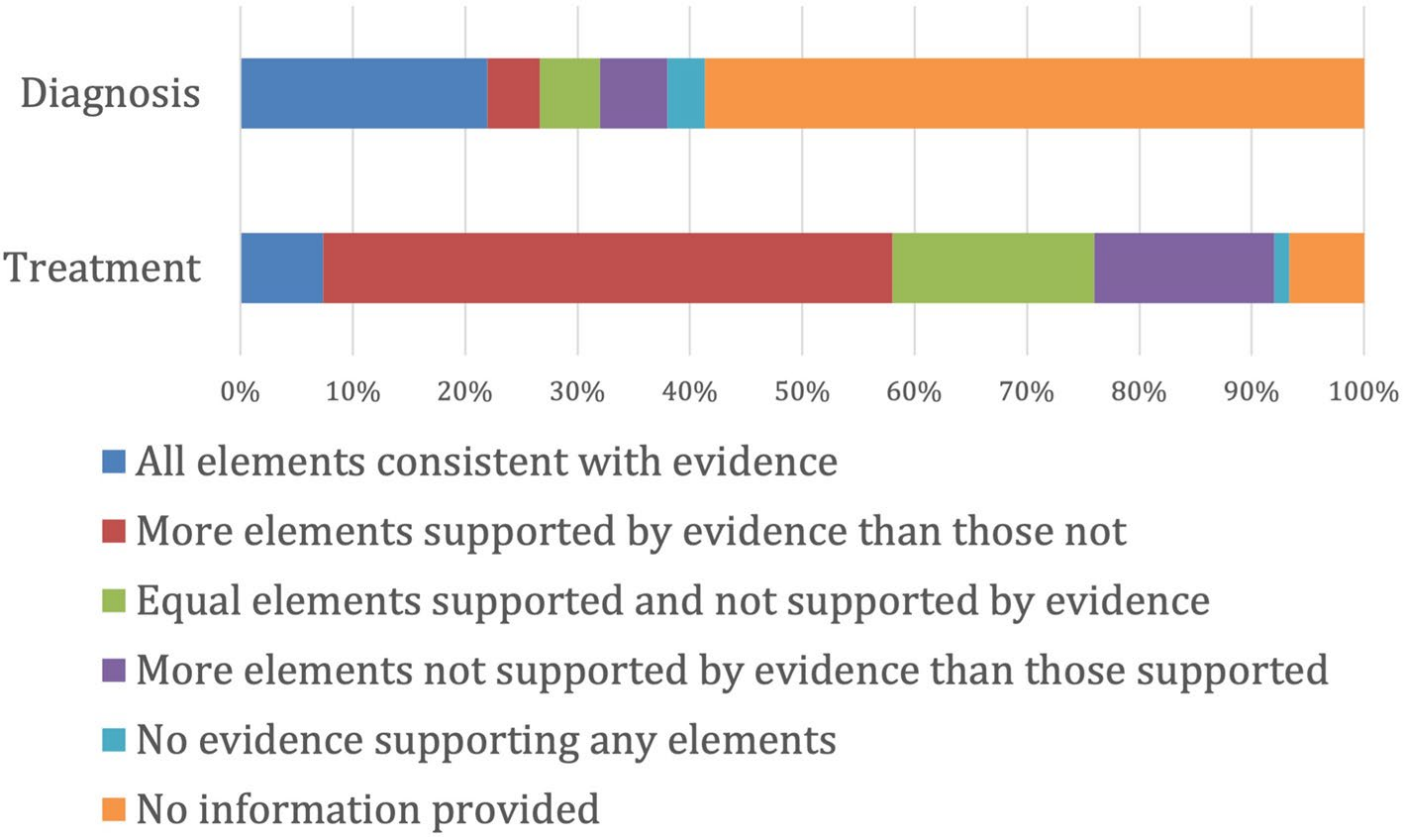
Diagnosis and treatment advertising elements alignment with evidence

Most websites described calcaneal apophysitis presenting in the age range 8 to 14 years, however there was a large variety of age ranges described with little to no referencing. Many websites (*n* = 88, 59%) did not provide any information about how calcaneal apophysitis was diagnosed, yet the majority provided at least one treatment recommendation (*n* = 140, 93%). There were 11 (7%) websites with all treatment recommendations having support of evidence (Fig. 1). There was a mean (SD) of 6 (3) treatments recommendations per website, with 18 websites providing nine or more treatment options. Detailed information of the grouping categories for different recommended treatments and the frequency of grouping categories can be found in Additional files 3 and 4.

## Discussion

This research about health information advertising, identified many health professionals provide information about a common childhood health condition that may not be understood by the target audience or not evidence based. The online information commonly lacked credibility elements such as date of publication or references and used multiple terms for the same conditions. The literacy levels required to understand the information was often higher than the recommended literacy level for the general public [26], and far higher than the population who commonly are diagnosed with this condition. Websites more frequently provided accurate diagnosis information than recommending evidence-based treatments. Some treatment options would result in high health care utilisation such as surgery. While some websites recommended interventions contraindicated in the age group, such as extracorporeal shock wave therapy [27], or recommended treatments with no evidence such as laser therapy. The way this condition was described, and treatment information was written in online advertising, has the potential to encourage health seeking behaviours due to fear and misinformation. It also has the potential to create confusion due to health professionals providing conflicting information, particularly with treatments that have little known effect or even serious consequences.

It was disappointing to find less than half the audited websites provided reassurance about the benign nature of this condition, and the majority still described it with its disease-based eponymous name [16]. There is a long-standing call for removal of eponymous names throughout healthcare, position papers calling for no publications including eponymous terms [28], with even Dr James Sever calling it calcaneal apophysitis in his seminal paper [16]. Despite this, it appears the Sever’s Disease naming convention is internationally entrenched in healthcare. This has the potential to increase parental and child fear by implying a debilitating condition that will cause severe harm [29] and always require healthcare assistance in pain resolution.

Healthcare seeking for benign conditions can be reduced through easily understood information [30]. It is also logical that information be targeted to the population more likely to present with this condition. This is particularly relevant as more teenagers seek online health information with increased computer literacy and access [31]. Despite this, the information was provided at a higher literacy level than those commonly impacted, with only 19 (13%) of websites aimed at the literacy level of older end of population likely to be impact by this condition. Literacy levels of these websites also potentially impact families. Across the audited countries, it is estimated 15.5% of adults have the literacy level of Level 1 or less [32]. This means, in practical terms, that the reader may not be able to determine the correct amount of medicine to give a child based on the information printed on the package [33]. The recommended readability level of health care information for all readers is grade eight level [26], which only 45 (30%) websites met.

We identified discrepancies in the recommended diagnosis method and, more frequently, in the management of calcaneal apophysitis. There was little consensus of the age of presentation, despite unequivocal evidence of the apophysis appearance between the ages of 5–14 [34]. We also found 40 different types or descriptions of treatments; a list unexpectedly so diverse we grouped into proposed mechanism of action to collate the results. We could not find supporting evidence for many of the treatments that websites recommended, such as stretching. It is unknown what impact this has on parents, their knowledge of the condition, fear or health seeking behaviours.

This is not the first study to identify concerns with accuracy of the paediatric health information advertised online [1]. Clinicians must be consistent and vigilant to address misinformation rather than contributing to the problem. Apophyseal injuries are more frequent in children who play sport, therefore some children may require tailored advice relating to their current sport and training requirements. However, this is not relevant to all children with apophyseal injuries, meaning there is potentially too much information provided for families without the clinical insight of health professionals to make good decisions about self-care or seeking care. When producing this online advice, it was also unknown what clinical oversight clinicians had for their websites, or if the disparities existed because of knowledge deficit, anecdotal evidence or not keeping up with contemporary practice [35]. The knock-on effect of this practice in this case could be unnecessary referrals, investigations, and an overuse of treatment prescriptions such as that seen in other benign conditions [36].

We acknowledge the limitations of the study. Our findings only apply to the first fifty articles from a Google search in each location and over time these articles may no longer be popular due to changes in the algorithm. Lower ranked websites may have presented different information. We used as many elements as possible from both the DISCERN tool [11] and JAMA instrument [12], but not both in their entirety. We strongly considered their individual use, but both had elements not relevant to the aims which meant limiting our findings. We were not able to identify any other tools to use in their entirety that were appropriate to our aim. Lastly, we acknowledge the challenge of grouping the treatment choices and that not all treatments included were not subject to assessment of risk of bias. Despite our groupings being based on consensus of external researchers and clinicians to the authorship of this paper, it has not been broadly tested via peer review. Therefore, we attempted a pragmatic and cautious approach, considering the vast number of recommended treatments. Two included clinical trials, while prospectively registered and published in peer reviewed journals, should be viewed as the level of evidence below that of a systematic review without meta-analysis. Lastly, despite this, it is unknown what translation of these diagnostic services or treatments recommended by health professionals for calcaneal apophysitis actually occur in actual practice.

Recent studies align patient expectations of additional (and often unnecessary) diagnostic imaging and treatment modalities or initiation of self-care strategies with the accuracy and quality of online health advertising information [37]. This highlights the opportunities clinicians have in providing quality advice for benign childhood condition information to have broader health care implications. The Choosing Wisely initiative is embedded in the health care systems of countries we audited [38]. This initiative focuses on improving health outcomes for people in partnership with their health care provider, while reducing health care wastage (reducing unnecessary diagnostic tests) and low value care. Our research highlights that it may be appropriate to introduce recommendations for conditions such as apophyseal injuries to increase consistency in advertising. In the meantime, clinicians could make simple changes to how they provide messaging to current or potential families of children with heel pain. This could be through using an easy-to-read English writing style, reviewing published evidence of high value and effective treatments, and then aligning public facing advertising to contemporary evidence. For example, encouraging temporarily reducing load (or sport types), or simple over the counter heel raisers and seek care where these do not reduce pain. Clinicians could also consider advertising simple treatment advice focused on home-based initiatives parents could commence prior to seeking care, thereby enabling tailored advice and treatment to child’s particular circumstances, activity level and training load to in clinic consultations when simple initiatives have not worked.

## Conclusion

Calcaneal apophysitis online advertising is mostly provided by clinicians and there are diagnostic and treatment modalities advertised that are not supported by evidence. More concerning was the number of low-value (high cost without high benefit), or inappropriate treatments being advertised that may place the child at unnecessary or unsubstantiated pain or infection. These treatments included laser therapy, extracorporeal shock wave therapy or Achilles tendon lengthening surgery. Clinicians should consider revising any online information to increase understandability and incorporating evidence-based treatments. These finding have practical application. That is, health professionals have an ethical responsibility to provide accurate and evidence-based online advertising, providing reassurance calcaneal apophysitis is a benign condition, when to seek care, and treatments that have evidence supporting their use. This research highlights the opportunities for health professionals to align diagnostic and treatment recommendations for calcaneal apophysitis with high value care. Health professionals also should regularly review their information to ensure it aligns with current evidence or point consumers to contemporary and regularly reviewed information.

## Supplementary Information

**Additional file 1: Appendix A.** Data extraction template.

**Additional file 2: Appendix 2.** Randomised list of audited websites and their country of origin.

**Additional file 3: Appendix 3.** Coding legend and coding corresponding to 150 websites for diagnosis and treatment elements where grey shading are elements without evidence supporting their use.

**Additional file 4: Appendix 4.** 150 websites coded against elements with and without evidence.

## Data Availability

All data generated are included in this published article and supplementary information files.

## Abbreviations

SD: Standard deviations
SMOG: Statistical Measure of Gobbledygook
UK: United Kingdom
USA: United States of America
