# Supplementary material for: International differences and inaccuracies in the public advertising about calcaneal apophysitis: an audit of websites originating in Australia, UK and USA
Source: J Foot Ankle Res. 2023 Jun 20;16:39. doi: 10.1186/s13047-023-00637-9 (PMC10280899; doi:10.1186/s13047-023-00637-9)
Supplement: Supplementary file 1 — Additional file 1: Appendix A. Data extraction template. [file 13047_2023_637_MOESM1_ESM.docx]

**Appendix A:** Data extraction template

| Information grouping | Data elements |
| --- | --- |
| **Credibility** |  |
| Website | URL |
| Hosting country | Australia |
|  | UK |
|  | USA |
| *Publication date* | Date |
| *Publisher* | Government entity |
|  | Public entity or consumer advocacy organisation |
|  | Health care service (e.g., hospital) |
|  | Private practice |
|  | Peak board (e.g., health professional association) |
|  | Media (e.g., Blog, news website) |
|  | Profession/s sponsoring the content or part of the site providing the information |
| **Readability** | SMOG score |
|  | Number of words |
|  | Percentage of complex words |
|  | Number of alternative supporting videos or images |
| **Accuracy relating to apophysis** | Naming convention, and order of naming convention where multiple names were used |
|  | Location consistent with diagnosis |
|  | Age group |
|  | Aetiology |
|  | How pain may be exacerbated |
|  | Red flags/co-diagnoses |
|  | Trajectory |
|  | Diagnosis methods |
|  | Treatment methods |
