# Supplementary material for: International differences and inaccuracies in the public advertising about calcaneal apophysitis: an audit of websites originating in Australia, UK and USA
Source: J Foot Ankle Res. 2023 Jun 20;16:39. doi: 10.1186/s13047-023-00637-9 (PMC10280899; doi:10.1186/s13047-023-00637-9)
Supplement: Supplementary file 2 — Additional file 2: Appendix 2. Randomised list of audited websites and their country of origin. [file 13047_2023_637_MOESM2_ESM.docx]

Appendix 2. Randomised list of audited websites and their country of origin.

| Website | Country |
| --- | --- |
| <https://www.betterhealth.vic.gov.au/health/conditionsandtreatments/severs-disease> | Australia |
| <http://www.podiatrycare.com.au/conditions-we-treat/heel-pain-in-children/> | Australia |
| <https://www.heelclinic.com.au/blog/heel-pain-in-kids/> | Australia |
| <https://www.foundationpodiatry.com.au/heel-pain-in-children-why-do-my-child-s-heels-hurt-when-playing-sport/> | Australia |
| <https://insteppodiatrybrisbane.com.au/severs-disease-common-cause-heel-pain-children/> | Australia |
| <https://posturepodiatry.com.au/severs-disease-treatment-that-really-does-help/> | Australia |
| <https://acpodiatry.com.au/3-tips-to-fixing-heel-pain-in-kids/> | Australia |
| <https://www.cddft.nhs.uk/media/794604/heel%20pain.pdf> | UK |
| <https://www.esht.nhs.uk/wp-content/uploads/2020/10/0846.pdf> | UK |
| <https://www.nhs.uk/conditions/foot-pain/heel-pain/> | UK |
| <https://cks.nice.org.uk/topics/developmental-rheumatology-in-children/management/heel-pain-in-children/> | UK |
| <https://www.physio.co.uk/what-we-treat/paediatric/conditions/children/heel-pain.php> | UK |
| <https://www.nuh.nhs.uk/severs-disease/> | UK |
| <http://www.londonorthopaedicsurgery.co.uk/children%E2%80%99s-foot-ankle/heel-pain-in-children/> | UK |
| <https://www.hct.nhs.uk/media/2478/severs-june-2017.pdf> | UK |
| <https://www.yorkhospitals.nhs.uk/childrens-centre/your-childs-hospital-journey/therapy-services/physiotherapy/heel-pain/> | UK |
| <https://southdevonfootclinic.co.uk/calcaneal-apophysitis/> | UK |
| <https://www.synergyphysio.co.uk/news/heel-pain-in-children-severs-disease> | UK |
| <https://www.mcht.nhs.uk/EasysiteWeb/getresource.axd?AssetID=23815&type=full&servicetype=Attachment> | UK |
| <https://footactive.co.uk/severs-disease> | UK |
| <https://www.elht.nhs.uk/download_file/2744/2575> | UK |
| <https://doclibrary-rcht.cornwall.nhs.uk/GET/d10338474> | UK |
| <https://www.uhd.nhs.uk/uploads/about/docs/our_publications/patient_information_leaflets/Childrens_therapy/Childrens_physiotherapy/heel_pain_updated_2019-1.pdf> | UK |
| <https://www.physio-pedia.com/Sever%27s_disease> | UK |
| <https://bhamfoot.com/severs-disease-a-common-cause-of-heel-pain-in-children/> | UK |
| <https://www.dailymail.co.uk/health/article-2528585/Sore-heel-thats-sign-childs-grown-fast-good.html> | UK |
| <https://podfitadelaide.com.au/severs-heel-pain/> | Australia |
| <https://www.proactivehm.com.au/heel-pain-in-kids/> | Australia |
| <https://www.footandleg.com.au/my-childs-heel-sore-severs-condition/> | Australia |
| <https://ryanfootandankleclinic.com/podiatry-services/childrens-foot-ankle-problems/severs-disease-childrens-heel-pain/> | USA |
| <https://www.rockymountainfootandanklecenter.com/blog/heel-pain-in-kids> | USA |
| <https://www.nationwidechildrens.org/-/media/documents/158489> | USA |
| <https://www.fixmyfoot.co.uk/severs-heel-windsor-maidenhead.html> | UK |
| <https://www.activestep.co.uk/2020/02/04/heel-pain-in-children-what-could-this-be/> | UK |
| <https://www.footcarescotland.co.uk/severs-disease/> | UK |
| <https://www.topdoctors.co.uk/medical-articles/sever-s-disease-what-need-know-heel-pain-children> | UK |
| <https://www.cspc.co.uk/complex-conditions/severs-disease-heel-pain/> | UK |
| <https://www.gavnoble.com/focus-on-severs-disease/> | UK |
| <https://hempsteadtherapycentre.co.uk/2020/02/21/severs-disease/> | UK |
| <https://bartholomewwayclinic.co.uk/severs-disease-treatment> | UK |
| <https://fitterfeet.co.uk/childrens-feet/> | UK |
| <https://www.davidjstock.co.uk/treatments/severs-disease/> | UK |
| <https://www.plymouthpodiatry.co.uk/sever-s-disease> | UK |
| <https://www.teamgrassroots.co.uk/severs-disease-symptoms-signs-and-treatments/> | UK |
| <https://www.alderbankphysio.co.uk/heel-pain-in-adolescents-severs-condition/> | UK |
| <https://www.chiropody.co.uk/what-we-treat/biomechanical-problems/the-ankle/severs-disease.php> | UK |
| <https://www.manchesterphysio.co.uk/what-we-treat/paediatric-physiotherapy/severs-disease.php> | UK |
| <https://www.jonwsportsinjury.co.uk/2020/10/26/growth-related-injuries-in-children-osgood-schlatter-disease-and-severs-disease/> | UK |
| <https://www.manchesterpodiatry.co.uk/services/paediatric-podiatry/foot-pain.php> | UK |
| <https://www.podogo.com/2018/02/09/child-see-podiatrist/> | UK |
| <https://zestpodiatry.co.uk/calcaneal-apophysitis/> | UK |
| <https://evolutionpodiatry.co.uk/?p=1188> | UK |
| <https://www.wsh.nhs.uk/CMS-Documents/Patient-leaflets/TraumaandOrthopaedics/6087-1-Severs-disease.pdf> | UK |
| <https://www.upandrunningpodiatry.com.au/heel-pain-kids-severs-disease/> | Australia |
| <https://www.profeetpodiatry.com.au/2020/09/how-to-reduce-heel-pain-in-children> | Australia |
| <https://brindabellapodiatry.com.au/heel-pain-in-your-child/> | Australia |
| <https://www.thefootcareclinic.com.au/severs-disease-what-is-severs-disease-and-how-can-i-help-my-child-with-heel-pain/> | Australia |
| <https://www.rch.org.au/uploadedFiles/Main/Content/rheumatology/SEVERS%20DISEASE%20VPON%20-%20PDF.pdf> | Australia |
| <https://www.myhealthteam.com.au/Podiatry/Children-s-Heel-Pain-Sever-s-Disease> | Australia |
| <https://podiatryonwilliamson.com.au/heel-pain-severs-disease/> | Australia |
| <https://www.sydneyheelpain.com.au/severs-disease-causes-symptoms-treatment/> | Australia |
| <https://www.fnqpodiatry.com.au/cairns-podiatry-orthotics-services/childrens-heel-pain-severs-treatment-cairns/> | Australia |
| <https://www.feetology.com.au/childrens-heel-pain/> | Australia |
| <https://www.psfootclinic.com.au/how-we-help/heel-pain-in-children-severs-disease> | Australia |
| <https://www.melbournepodiatryclinic.net.au/suffer-from-heel-pain/> | Australia |
| <https://www.dynamicpodiatry.com.au/heel-pain-in-children-severs-apophysitis/> | Australia |
| <https://www.healthdirect.gov.au/severs-disease> | Australia |
| <http://www.malvernpodiatrysurgery.co.uk/conditions/severs-disease.html> | UK |
| <https://www.feetinmotion.co.uk/blog/severs-disease/> | UK |
| <https://solemaids.co.uk/heel-pain-in-childrens-feet/> | UK |
| <https://www.fitbyphysio.com/severs> | UK |
| <https://sportandspinalphysio.com.au/fix-heel-pain-severs-disease-in-children/> | Australia |
| <https://www.13thbeachhealthservices.com.au/severs-disease-heel-pain/> | Australia |
| <https://www.thephysiodepot.com.au/severs-disease-heel-pain-in-kids/> | Australia |
| <https://www.pioneerpodiatry.com.au/heel-pain-in-children-severs> | Australia |
| <https://www.outoftheboxphysio.com.au/post/2017/02/23/heel-pain-in-kids> | Australia |
| <https://onepointhealth.com.au/physiotherapy/childrens-heel-pain-severs-disease/> | Australia |
| <https://www.fairfieldpodiatry.com.au/blog/2018/8/6/heel-pain-in-children> | Australia |
| <https://bankstownpodiatry.com.au/severs-disease/> | Australia |
| <https://www.rockinghampodiatry.com.au/childrens-feet/children-heel-pain/> | Australia |
| <https://www.tfcpodiatry.com.au/severs-disease-a-cause-of-heel-pain-in-children/> | Australia |
| <https://www.perthpodiatricsurgery.com/severs-disease-how-can-i-help-my-child-with-heel-pain/> | Australia |
| <https://physio4all.com.au/does-your-child-have-heel-pain/> | Australia |
| <https://footlogics-shop.com.au/foot-complaints/severs-disease/> | Australia |
| <https://runningscience.com.au/does-your-child-have-heel-pain> | Australia |
| <https://www.baysidesportspodiatry.com.au/news/2021/3/24/my-child-has-heel-pain> | Australia |
| <https://www.footrightpodiatry.com.au/conditions/kids-heel-pain-calcaneal-apophysitis> | Australia |
| <https://www.beyondpodiatry.com.au/heel_pain_plantar_fasciitis_heel_spurs_severs_disease.html> | Australia |
| <https://www.melbournepodiatristsandorthotics.com.au/conditions/childrens-feet/severs-disease/> | Australia |
| <https://www.acfas.org/Content.aspx?id=1483> | USA |
| <https://pediatricfootankle.com/foot-conditions/pediatric-heel-pain/> | USA |
| <https://www.foothealthfacts.org/article/don-t-ignore-your-kid-s-heel-pain> | USA |
| <https://kidshealth.org/en/parents/severs-disease.html> | USA |
| <https://www.bouldermedicalcenter.com/dont-dismiss-your-childs-heel-pain/> | USA |
| <https://www.healthline.com/health/parenting/heel-pain-in-kids> | USA |
| <https://www.childrenshospital.org/conditions-and-treatments/conditions/s/severs-disease> | USA |
| <https://orthoinfo.aaos.org/en/diseases--conditions/severs-disease/> | USA |
| https://health.clevelandclinic.org/has-heel-pain-sidelined-your-child-7-questions-on-severs-disease-answered/ | USA |
| https://www.montrosefootdoctor.com/contents/patient-education/medical-conditions/pediatric-heel-pain | USA |
| <https://www.kalmarfamilypodiatry.com/blog/is-your-child-complaining-of-heel-pain> | USA |
| <https://healthcare.utah.edu/the-scope/shows.php?shows=1_6a8zujkb> | USA |
| <https://www.healthychildren.org/English/health-issues/injuries-emergencies/sports-injuries/Pages/Heel-Pain-and-Severs-Disease.aspx> | USA |
| <https://familydoctor.org/condition/severs-disease/> | USA |
| <https://www.pafootdoctors.com/our-specialties/pediatric/heel-pain-in-children> | USA |
| <https://www.stlouischildrens.org/health-resources/pulse/heel-pain-young-athletes-severs-disease> | USA |
| <http://www.yourfamilyfootcare.com/library/a-cause-behind-childrens-heel-pain.cfm> | USA |
| <https://www.ankle-footspecialist.com/blog/heel-pain-and-your-child-what-you-should-know> | USA |
| <https://lifespanosteopathy.com.au/heel-pain-in-pre-teen-kids/> | Australia |
| <http://sportsmedosteo.com.au/heel-pain-severs-disease/> | Australia |
| <https://www.riversidepodiatry.com.au/heel-pain-in-children-severs-disease/> | Australia |
| <https://ankleandfootcentre.com.au/2019/01/case-study-13-heel-pain-child/> | Australia |
| <https://watsoniapodiatry.com.au/heel-pain-in-kids-what-does-it-mean/> | Australia |
| <https://www.sportnova.co.uk/severs-disease> | UK |
| <https://www.ghc.nhs.uk/wp-content/uploads/doc_download/2019/Childrens_Physio/FS43_Heel_Pain_Jun_2018.pdf> | UK |
| <https://waldegraveclinic.co.uk/aches-pains-in-sporty-kids-explained/> | UK |
| <https://www.one2onetherapy.net/severs-disease/> | UK |
| <https://www.backtoback432.co.uk/back-to-back-blog/severs-disease-heel-pain-in-children> | UK |
| <https://www.londonorthotics.co.uk/orthotics/paediatric/severs-disease/> | UK |
| <https://wacopodiatry.com/childrens-heel-pain-severs-disease/> | USA |
| <https://www.corriganpodiatry.com/pediatric-heel-pain/> | USA |
| <https://socalfootandankle.com/child-heel-pain/> | USA |
| <https://justfootankle.com/severs-disease-heel-pain-kids/> | USA |
| <https://www.queencityfootandankle.com/blog/understanding-your-childs-heel-pain> | USA |
| <https://www.active.com/soccer/articles/how-to-deal-with-your-child-s-heel-pain-881853> | USA |
| <https://www.hmpgloballearningnetwork.com/site/podiatry/article/2350> | USA |
| <https://kidspluspgh.com/doctors-notes/heel-pain/> | USA |
| <https://www.martinfootandankle.com/practice_areas/severs-disease.cfm> | USA |
| <https://gentlefootcareoh.com/heel-pain-in-children/> | USA |
| <https://gotofootdoc.com/services/childrens-foot-care/severs-disease/> | USA |
| <https://ottawafootclinic.com/childrens-heel-pain/> | USA |
| <https://inspirepodiatry.com.au/heel-pain-in-children/> | Australia |
| <https://entirepodiatry.com.au/expertise/children/heel-pain/> | Australia |
| <https://www.wellheeledpodiatry.com.au/severs-disease> | Australia |
| <https://countryfootcare.com/library/childrens-feet/severs-disease-common-heel-pain-in-children/> | USA |
| <https://www.kazmerfootandanklecenters.com/blog/look-for-these-signs-to-see-if-your-child-has-heel-pain> | USA |
| <https://bhamfoot.com/severs-disease-a-common-cause-of-heel-pain-in-children/> | USA |
| <http://www.southfloridasportsmedicine.com/child-severs-heel-pain.html> | USA |
| <https://illinoisfoot.com/foot-pathologies/pediatrics/heel-pain/> | USA |
| <https://familyfootcareofjasper.com/childrens-foot-care/severs-disease/> | USA |
| <https://austinfootandankle.com/conditions/severs-disease/> | USA |
| <https://scottishriteforchildren.org/news-items/does-your-child-complain-of-heel-pain-during> | USA |
| <https://www.verywellhealth.com/heel-pain-in-children-and-adolescents-1337746> | USA |
| <https://kansasfootclinic.com/how-to-help-your-active-child-recover-quickly-from-heel-pain/> | USA |
| <https://www.inpodiatrygroup.com/faqs/do-you-know-what-your-childs-heel-pain-could-mean.cfm> | USA |
| <https://www.ortho.wustl.edu/content/Patient-Care/3189/Services/Pediatric-and-Adolescent-Orthopedic-Surgery/Overview/Knee-Education-Overview/Severs-Disease.aspx> | USA |
| <https://barnesfootandankle.com/pediatric-heel-pain/> | USA |
| <https://www.stanfordchildrens.org/en/topic/default?id=sever-disease-in-children-160-42> | USA |
| <https://www.webmd.com/children/severs-disease-kids-teens> | USA |
| <https://www.elitefootankle.com/docs/4056.pdf> | USA |
| <https://www.elmhurstfootdoc.com/blog/everything-you-need-to-know-about-childhood-heel-pain.cfm> | USA |
