# Supplementary material for: International differences and inaccuracies in the public advertising about calcaneal apophysitis: an audit of websites originating in Australia, UK and USA
Source: J Foot Ankle Res. 2023 Jun 20;16:39. doi: 10.1186/s13047-023-00637-9 (PMC10280899; doi:10.1186/s13047-023-00637-9)
Supplement: Supplementary file 3 — Additional file 3: Appendix 3. Coding legend and coding corresponding to 150 websites for diagnosis and treatment elements where grey shading are elements without evidence supporting their use. [file 13047_2023_637_MOESM3_ESM.docx]

Appendix 3. Coding legend and coding corresponding to 150 websites for diagnosis and treatment elements where grey shading are elements without evidence supporting their use.

| Treatment element combined into a grouping | Treatment modalities in grouping |
| --- | --- |
| Load reduction strategies | Load/activity modification, rest, activity reduction but not cessation |
| Complete immobilisation | Casting, cam-boot, night immobilisation splint, use of crutches |
| Stretching | Recommendation for active or passive stretches performed by child |
| Exercise focused on building strength | This may include provision of physical therapy focused interventions such as eccentric strengthening exercises, gait retraining, pre/post training activities that increase muscle strength |
| Rest or reassurance | Pain science including reassurance, education about benign nature of condition, condition resolves with time |
| Pharmaceutical interventions | Non-steroidal anti-inflammatories, paracetamol, acetaminophen, injectable corticosteroids, topical non-steroidal anti-inflammatories or rubefacients, ice therapy |
| Footwear or heelcushioning | Footwear, gel pads, heel cushioning or padding, avoiding barefoot walking, changing training surfaces |
| Heel lifts | Device in footwear that lifts the heel |
| Ortho-mechanical interventions such as orthoses, bracing or taping | Prefabricated orthoses, custom made orthoses, braces or taping the ankle and foot. |
| Manual therapy | Massage, foam roller, foot mobilisation, dry needling |
| Other | Surgery to lengthen calf muscle or Achilles Tendon, extracorporeal shockwave therapy (ESWT), Laser therapy, weight management, electrical stimulation |
