# Supplementary material for: International differences and inaccuracies in the public advertising about calcaneal apophysitis: an audit of websites originating in Australia, UK and USA
Source: J Foot Ankle Res. 2023 Jun 20;16:39. doi: 10.1186/s13047-023-00637-9 (PMC10280899; doi:10.1186/s13047-023-00637-9)
Supplement: Supplementary file 4 — Additional file 4: Appendix 4. 150 websites coded against elements with and without evidence. [file 13047_2023_637_MOESM4_ESM.docx]

Appendix 4. 150 websites coded against elements with and without evidence (shaded elements coded as not having supporting evidence)

| Diagnosis elements | | | | | | Treatment elements | | | | | | | | | | |
| --- | --- | --- | --- | --- | --- | --- | --- | --- | --- | --- | --- | --- | --- | --- | --- | --- |
| Diagnosis through history taking | Diagnosis through physical examination | Diagnosis through x-ray | Diagnosis through other imaging such as CT scan or MRI | Advised imaging not commonly required | Diagnosis through pathology testing | Load reduction strategies | Complete immobilisation | Stretching | Exercise/ focused on building strength | Rest/reassurance | Pharmacological interventions (oral, topical or injected) | Footwear/ heel cushioning | Heel lifts | Ortho-mechanical interventions such as orthoses, bracing or taping | Other including ESWT*, surgery, laser | Manual therapies |
| 1 | 1 |  |  | 1 |  | 3 |  |  |  |  | 1 | 2 |  |  |  |  |
|  |  |  |  |  |  | 3 |  | 1 |  |  |  | 2 |  |  |  |  |
|  |  |  |  |  |  | 1 |  | 1 |  |  | 1 | 1 |  | 1 |  |  |
|  |  |  |  |  |  |  |  |  |  |  |  |  |  |  |  |  |
|  |  |  |  |  |  | 2 |  | 1 | 1 |  |  |  | 1 | 2 |  |  |
| 1 | 1 |  |  | 1 |  | 1 |  | 1 |  |  | 1 |  |  | 1 |  |  |
| 1 | 1 |  |  | 1 |  | 1 |  |  | 1 | 1 |  | 1 |  |  | 1 |  |
|  |  |  |  |  |  | 2 |  | 2 |  |  | 2 | 1 | 1 | 1 |  |  |
|  |  |  |  |  |  | 2 |  | 1 |  |  |  | 2 |  |  |  |  |
| 1 | 1 |  |  | 1 |  |  | 1 | 1 | 1 |  | 2 | 2 | 1 | 2 |  | 1 |
|  |  |  |  |  |  |  |  |  |  |  |  |  |  |  |  |  |
|  |  |  |  |  |  | 1 |  | 1 |  | 1 | 2 | 1 |  |  |  |  |
|  |  |  |  |  |  | 1 | 1 | 1 |  | 1 | 2 | 1 |  | 1 |  |  |
|  |  |  |  |  |  | 1 |  | 1 |  | 1 | 2 | 1 |  | 1 |  |  |
|  |  |  |  |  |  | 1 |  | 1 |  |  | 2 |  |  | 1 |  |  |
|  |  |  |  |  |  | 2 |  | 1 |  |  | 1 | 1 |  | 2 |  |  |
| 1 | 1 |  |  | 1 |  | 2 |  | 1 |  |  | 2 |  | 1 | 3 |  |  |
|  |  |  |  |  |  |  |  | 1 |  |  | 2 |  |  | 2 |  |  |
|  |  |  |  |  |  |  |  | 1 |  |  | 1 | 1 | 1 | 1 |  |  |
|  |  |  |  |  |  | 1 | 1 | 1 | 1 |  | 2 | 1 |  | 1 |  |  |
|  |  |  |  |  |  | 2 |  |  |  |  | 1 | 2 | 1 |  |  |  |
|  |  |  |  |  |  | 1 |  | 2 |  | 1 | 1 | 1 |  |  |  |  |
|  |  |  |  |  |  |  |  | 1 |  | 1 |  |  |  |  |  |  |
| 1 | 1 |  |  |  |  | 1 |  | 1 | 3 |  | 1 |  |  | 1 |  |  |
|  |  |  |  |  |  | 1 |  |  |  | 1 |  |  |  | 1 |  |  |
| 1 | 1 |  |  |  |  | 2 |  |  | 1 |  | 1 | 1 | 1 | 2 |  |  |
|  |  |  |  |  |  | 1 |  | 1 |  |  | 1 |  |  | 1 |  |  |
|  |  |  |  |  |  |  |  |  |  |  |  |  |  |  |  |  |
| 1 | 1 | 1 | 1 |  |  |  | 1 | 1 |  | 1 |  |  |  | 2 |  |  |
| 1 | 1 |  |  |  |  | 1 |  |  |  |  | 1 | 1 | 1 | 2 |  |  |
|  |  |  |  |  |  | 1 |  |  | 1 |  | 1 | 1 |  |  |  |  |
| 1 | 1 |  |  | 1 |  | 1 | 1 | 1 |  |  | 2 |  |  | 1 |  |  |
|  |  |  |  |  |  | 1 |  | 1 | 1 |  |  | 1 | 1 | 2 |  |  |
|  |  |  |  |  |  | 1 |  | 1 |  |  |  | 1 |  | 1 |  |  |
|  |  |  |  |  |  |  |  |  |  |  |  |  |  | 1 |  |  |
|  |  |  |  |  |  | 1 |  | 1 | 1 |  | 1 |  | 1 | 1 |  |  |
| 1 | 1 |  |  |  |  |  |  | 1 |  | 1 | 1 | 1 |  | 1 |  |  |
| 1 | 1 |  |  | 1 |  | 1 | 2 | 1 | 1 |  | 3 |  | 1 |  |  |  |
|  |  |  |  |  |  | 1 |  | 1 |  |  | 1 |  | 1 |  |  |  |
|  |  |  |  |  |  |  |  | 1 |  |  |  |  |  |  |  |  |
|  |  |  |  |  |  | 1 | 1 | 1 | 1 |  | 2 | 1 |  | 1 |  |  |
| 1 | 1 |  |  | 1 |  | 1 |  | 1 | 1 |  | 2 | 1 | 1 |  |  |  |
| 1 | 1 | 1 |  |  |  | 1 |  |  |  | 1 | 1 |  | 1 | 1 |  |  |
|  |  |  |  |  |  | 1 |  | 1 | 1 |  |  | 1 | 1 |  |  |  |
|  |  |  |  |  |  | 1 |  | 1 |  |  | 1 |  |  |  |  |  |
|  |  |  |  |  |  |  |  |  |  | 1 |  |  |  |  |  |  |
|  |  |  |  |  |  |  |  |  |  |  |  |  |  |  |  |  |
|  |  |  |  |  |  |  |  | 1 |  |  | 1 | 1 | 1 | 1 |  |  |
| 1 | 1 |  |  | 1 |  | 1 |  |  |  |  | 1 | 1 |  | 1 |  |  |
| 1 | 1 |  |  |  |  | 1 |  | 1 |  | 1 | 1 | 2 |  |  |  |  |
| 1 | 1 | 1 |  |  |  | 1 |  |  |  | 2 | 2 | 1 | 1 | 1 |  |  |
|  |  |  |  |  |  |  |  | 1 | 1 |  | 1 | 1 |  | 1 |  |  |
| 1 | 1 | 1 |  |  |  | 1 |  | 1 | 1 | 1 | 2 | 2 | 1 |  |  |  |
|  |  |  |  |  |  | 1 |  |  | 1 |  | 1 | 3 |  | 1 |  | 1 |
|  |  |  |  |  |  | 2 | 1 | 1 |  | 1 | 2 | 4 | 1 | 2 |  |  |
|  | 1 | 1 | 1 |  |  | 1 |  | 1 | 2 |  | 4 | 2 |  | 2 |  |  |
|  |  |  |  |  |  |  |  |  |  |  |  | 1 | 1 | 1 |  |  |
|  |  |  |  |  |  | 2 | 1 | 1 |  |  |  | 1 |  | 1 |  |  |
|  |  |  |  |  |  | 1 |  | 1 | 1 | 1 | 2 | 1 |  | 1 |  |  |
|  | 1 | 1 | 1 |  |  |  |  |  |  |  |  |  | 1 | 1 |  |  |
|  | 1 |  |  |  |  | 1 |  | 1 | 1 |  | 1 | 1 | 1 | 2 |  |  |
|  | 1 |  |  |  |  | 1 |  | 1 |  | 1 | 1 | 1 |  | 1 |  |  |
|  |  |  |  |  |  |  |  | 1 | 1 |  | 2 | 1 |  |  |  | 2 |
|  |  |  |  |  |  |  |  |  |  |  |  |  |  |  |  |  |
|  |  |  |  |  |  |  |  | 1 |  | 1 | 1 | 1 | 1 | 2 |  |  |
|  | 1 |  |  |  |  |  |  | 1 |  |  | 2 | 2 | 1 | 2 |  |  |
|  |  |  | 1 |  |  | 1 |  | 1 | 1 |  | 1 | 1 | 1 | 1 | 1 | 1 |
|  |  |  |  |  |  | 1 |  |  | 1 | 1 |  | 1 |  | 1 |  |  |
|  |  |  |  |  |  | 1 |  | 1 | 1 |  | 1 | 1 | 1 | 1 |  | 1 |
|  |  |  |  |  |  | 1 |  |  |  | 1 |  |  |  |  |  |  |
|  | 1 |  |  |  |  |  |  | 1 |  |  | 1 | 2 | 1 | 1 |  |  |
| 1 | 1 | 1 | 1 |  |  |  | 1 |  | 2 | 1 |  | 1 | 1 | 2 |  | 1 |
|  | 1 |  |  |  |  | 1 |  |  |  |  |  | 1 |  | 1 |  |  |
|  |  |  |  |  |  |  |  | 1 |  |  | 2 |  | 1 |  |  |  |
|  |  |  |  |  |  | 1 |  | 1 |  |  | 2 |  | 1 |  |  |  |
|  |  |  |  |  |  |  |  | 1 |  | 1 | 1 |  | 1 | 2 |  |  |
| 1 | 1 |  |  |  |  |  |  | 1 |  | 1 | 1 | 2 |  |  |  |  |
|  | 1 |  |  |  |  |  |  | 1 | 1 | 1 | 2 | 1 | 1 | 2 |  | 2 |
|  | 1 |  |  |  |  |  |  | 1 |  |  | 1 | 2 |  | 2 |  | 2 |
|  |  |  |  |  |  | 1 |  | 1 |  |  | 2 | 1 |  | 1 |  | 1 |
|  |  |  |  |  |  | 1 |  | 1 |  |  | 2 | 3 |  | 1 |  | 1 |
|  |  |  |  |  |  | 1 |  | 1 | 1 | 1 | 1 |  |  |  |  |  |
| 1 |  | 1 |  |  |  | 1 | 1 |  | 1 |  | 1 | 1 |  | 2 |  |  |
|  |  |  |  |  |  |  |  |  |  |  |  |  |  |  |  |  |
|  |  |  |  |  |  | 1 | 1 |  |  | 1 |  | 1 | 1 | 2 | 1 | 2 |
|  | 1 | 1 |  |  |  |  |  | 1 |  | 1 | 1 |  |  |  |  |  |
|  |  |  |  |  |  | 1 |  |  | 1 |  | 2 | 2 |  | 2 |  |  |
|  |  |  |  |  |  | 1 | 1 | 1 |  | 1 | 1 |  | 1 | 1 |  | 1 |
|  |  |  | 1 |  |  | 1 | 1 | 1 |  |  |  | 3 | 1 | 1 |  |  |
|  | 1 |  |  |  |  | 1 |  | 1 | 1 |  | 1 | 1 | 1 | 2 |  | 1 |
|  |  |  |  |  |  |  |  |  |  |  |  | 1 |  | 1 |  |  |
|  | 1 |  |  |  |  |  |  | 1 | 2 |  |  | 1 |  | 1 |  |  |
|  |  |  |  |  |  |  |  | 1 |  |  |  | 1 | 1 | 1 | 1 |  |
|  |  |  |  |  |  | 1 |  | 1 | 1 |  | 2 | 1 | 1 | 2 |  | 2 |
|  |  |  |  |  |  | 1 |  | 1 |  | 1 | 1 |  |  | 1 |  |  |
| 1 | 1 |  |  |  |  |  |  | 1 | 3 |  | 2 | 2 | 1 | 1 |  | 1 |
|  | 1 |  |  |  |  | 2 |  | 1 |  |  | 2 | 1 | 1 | 1 |  | 1 |
|  |  |  |  |  |  | 1 |  |  |  |  |  | 1 | 1 | 1 |  |  |
|  |  |  |  |  |  |  |  | 1 |  | 1 | 1 |  |  | 1 | 1 |  |
|  |  |  |  |  |  |  |  | 1 | 1 |  |  | 1 |  | 1 |  |  |
| 1 | 1 | 1 | 1 |  | 1 | 1 | 1 | 1 | 1 |  | 1 |  |  | 2 |  |  |
|  |  | 1 |  |  |  | 1 | 2 |  |  |  | 1 | 1 |  | 1 |  |  |
|  |  |  |  |  |  | 2 |  |  | 1 |  | 1 | 1 | 1 |  |  |  |
| 1 | 1 |  |  | 1 |  |  | 1 |  | 1 |  | 2 | 2 |  | 2 |  |  |
| 1 |  |  |  | 1 |  |  | 1 | 1 | 1 |  | 1 |  |  | 1 |  |  |
|  | 1 |  |  |  |  |  |  | 1 |  |  | 2 |  | 1 |  |  |  |
|  | 1 | 1 |  |  |  | 1 |  | 1 | 1 |  | 2 | 1 |  |  |  |  |
|  | 1 |  |  |  |  |  | 1 | 1 |  |  | 1 | 2 | 1 |  |  |  |
|  |  |  |  |  |  | 1 | 1 |  |  | 1 | 1 | 2 |  |  |  |  |
| 1 | 1 | 1 | 1 |  | 1 | 1 | 1 |  | 1 |  | 1 |  |  | 2 | 1 |  |
|  |  |  |  |  |  | 2 | 1 | 1 | 2 | 2 | 1 | 1 | 1 | 2 |  |  |
|  |  |  |  |  |  |  | 1 | 1 | 1 | 1 | 2 | 2 |  |  |  |  |
|  | 1 | 1 |  |  |  | 1 |  | 1 |  | 1 | 2 | 2 | 1 |  |  |  |
|  |  |  |  |  |  |  |  | 1 | 1 | 1 | 2 | 2 |  | 1 |  |  |
|  |  |  |  |  |  | 1 |  |  | 1 |  | 1 | 1 |  | 1 |  |  |
|  | 1 | 1 |  |  |  | 1 | 1 | 1 |  | 1 | 1 | 1 |  |  |  |  |
|  |  |  |  |  |  | 2 |  | 1 |  | 1 | 1 | 1 |  | 1 |  |  |
|  |  |  |  |  |  | 1 |  | 1 |  | 1 | 1 | 1 |  |  |  |  |
|  | 1 | 1 | 1 |  | 1 |  | 1 | 1 |  | 1 | 1 | 1 |  | 1 | 1 |  |
|  | 1 | 1 | 1 |  | 1 |  |  |  |  |  |  |  |  |  |  |  |
|  | 1 |  |  |  |  | 1 | 1 | 1 |  | 1 |  | 1 |  |  |  |  |
|  |  |  |  |  |  |  |  | 1 | 1 | 1 | 1 | 1 |  | 1 |  |  |
|  |  |  |  |  |  |  |  | 1 |  | 1 | 1 | 1 |  |  |  |  |
|  |  |  |  |  |  |  |  | 1 | 1 |  | 1 | 2 |  |  |  |  |
| 1 | 1 | 1 | 1 |  | 1 | 1 |  | 1 | 1 |  |  |  | 1 | 1 |  |  |
|  |  |  |  |  |  | 1 |  | 1 | 1 | 1 | 1 | 1 |  |  |  |  |
|  |  |  |  |  |  |  | 1 | 1 |  |  | 2 | 2 |  | 1 |  |  |
|  |  |  |  |  |  |  |  |  |  | 1 | 1 | 2 |  |  |  |  |
|  |  |  |  |  |  |  |  |  |  | 1 |  |  |  | 1 |  |  |
|  | 1 |  |  |  |  |  | 1 | 1 |  |  |  |  |  | 1 |  |  |
|  |  |  |  |  |  |  | 1 | 1 |  |  | 1 |  |  | 1 |  |  |
|  | 1 | 1 |  |  |  |  |  |  |  | 1 | 1 | 2 |  | 2 |  |  |
|  |  |  |  |  |  |  |  | 1 |  | 1 | 2 | 1 |  |  |  |  |
|  |  | 1 |  |  |  | 1 |  | 1 |  |  | 1 | 1 | 1 | 1 |  |  |
|  |  |  |  |  |  |  |  | 1 |  | 1 | 1 | 1 |  |  |  |  |
|  |  |  |  |  |  |  |  | 1 |  |  | 2 |  |  | 1 |  |  |
|  | 1 |  |  |  |  | 1 | 1 |  |  |  |  | 1 | 1 | 2 |  |  |
| 1 | 1 | 1 | 1 |  | 1 | 1 | 1 | 1 | 1 |  | 1 |  |  | 1 |  |  |
|  |  |  |  |  |  | 2 | 1 | 1 |  |  | 2 |  |  |  |  |  |
| 1 | 1 | 1 |  |  |  | 1 | 1 |  | 1 |  | 1 |  |  | 2 |  |  |
|  |  |  |  |  |  |  |  |  |  | 1 | 1 | 2 |  |  |  |  |
|  |  |  |  |  |  |  |  | 1 | 1 | 1 | 1 | 1 |  |  |  |  |
|  |  |  |  |  |  |  |  | 1 |  | 1 | 1 | 1 |  | 1 | 1 |  |
|  |  |  |  |  |  |  |  |  |  |  |  |  |  |  |  |  |
|  |  | 1 |  |  |  | 1 |  | 1 |  |  | 2 | 1 |  |  |  |  |
|  | 1 |  | 1 |  | 1 | 1 | 1 |  |  |  | 1 | 1 |  | 1 | 2 |  |
| 1 | 1 |  |  |  |  | 1 |  |  |  |  | 2 | 1 |  | 1 |  |  |
|  | 1 |  |  |  |  | 1 |  | 1 |  | 1 | 1 | 1 |  | 1 |  |  |
|  |  |  |  |  |  | 1 | 1 | 1 | 1 |  | 1 |  |  | 1 | 1 |  |
| 1 |  | 1 |  |  |  |  | 1 | 1 |  |  | 2 | 2 |  |  |  |  |
